# Aluminium cyclo­hexa­phosphate

**DOI:** 10.1107/S1600536810005374

**Published:** 2010-02-13

**Authors:** Abdelghani Oudahmane, Aïcha Mbarek, Malika El-Ghozzi, Daniel Avignant

**Affiliations:** a Laboratoire de Chimie du Solide Minéral, Département de Chimie, Faculté des Sciences Semlalia, Université Cadi Ayyad, Marrakech, Morocco; b Laboratoire de Chimie Industrielle, Département de Génie des Matériaux, Ecole Nationale d’Ingénieurs de Sfax, Université de Sfax, BP W 3038, Sfax, Tunisia; c Laboratoire des Matériaux Inorganiques, UMR CNRS 6002, Université Blaise Pascal, 24 Avenue des Landais, 63177 Aubière, France

## Abstract

Single crystals of the title compound, Al_2_P_6_O_18_, were obtained by solid-state reaction. The monoclinic structure is isotypic with its Cr^III^, Ga^III^ and Ru^III^ analogues and is built up of six-membered phosphate ring anions, P_6_O_18_^6−^, isolated from each other and further linked by isolated AlO_6_ octa­hedra by sharing corners. Each AlO_6_ octa­hedron is linked to four P_6_O_18_^6−^ rings. More accurately, two rings are linked through bidentate diphosphate groups attached in the *cis*-positions to the AlO_6_ octa­hedron. The other two rings are linked to the two remaining corners, also in *cis*-positions of the AlO_6_ octa­hedron.

## Related literature

The title compound was first synthesized by Kanene *et al.* (1985 ▶) and its unit cell determined from Weissenberg photographs. Isotypic compounds have been reported: Ga_2_P_6_O_18_ (Chudinova *et al.*, 1987 ▶); Cr_2_P_6_O_18_ (Bagieu-Beucher & Guitel, 1977 ▶) and Ru_2_P_6_O_18_ (Fukuoka *et al.*, 1995 ▶). For a review of the crystal chemistry of cyclo­hexa­phosphates, see: Durif (1995 ▶, 2005 ▶). For applications of aluminium phosphate, see: Vippola *et al.* (2000 ▶). For the structures of other cyclo­hexa­phosphates with the P_6_O_18_^6−^ anion, see: Averbuch-Pouchot & Durif (1991*a* ▶,*b* ▶,*c* ▶).

## Experimental

### 

#### Crystal data

Al_2_P_6_O_18_
*M* *_r_* = 527.79
Monoclinic, 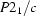

*a* = 6.0931 (2) Å
*b* = 15.0676 (4) Å
*c* = 8.2016 (3) Å
β = 105.166 (1)°
*V* = 726.75 (4) Å^3^
*Z* = 2
Mo *K*α radiation
μ = 0.96 mm^−1^
*T* = 296 K
0.16 × 0.07 × 0.06 mm

#### Data collection

Bruker APEXII CCD diffractometer
Absorption correction: multi-scan (*SADABS*; Bruker, 2008 ▶) *T*_min_ = 0.860, *T*_max_ = 0.945
6548 measured reflections
1674 independent reflections
1398 reflections with *I* > 2σ(*I*)
*R*_int_ = 0.035

#### Refinement

*R*[*F* ^2^ > 2σ(*F* ^2^)] = 0.032
*wR*(*F* ^2^) = 0.130
*S* = 1.16
1674 reflections
118 parameters
Δρ_max_ = 0.56 e Å^−3^
Δρ_min_ = −0.71 e Å^−3^

### 

Data collection: *APEX2* (Bruker, 2008 ▶); cell refinement: *SAINT* (Bruker, 2008 ▶); data reduction: *SAINT*; program(s) used to solve structure: *SHELXS97* (Sheldrick, 2008 ▶); program(s) used to refine structure: *SHELXL97* (Sheldrick, 2008 ▶); molecular graphics: CaRine (Boudias & Monceau, 1998 ▶) and *ORTEP-3* (Farrugia, 1997 ▶); software used to prepare material for publication: *SHELXTL*.

## Supplementary Material

Crystal structure: contains datablocks I, global. DOI: 10.1107/S1600536810005374/wm2303sup1.cif

Structure factors: contains datablocks I. DOI: 10.1107/S1600536810005374/wm2303Isup2.hkl

Additional supplementary materials:  crystallographic information; 3D view; checkCIF report

## Figures and Tables

**Table:** 

P1—O3	1.471 (2)
P1—O5	1.478 (2)
P1—O7	1.587 (2)
P1—O9	1.594 (3)
P2—O4	1.482 (2)
P2—O6	1.487 (2)
P2—O7^i^	1.579 (2)
P2—O8	1.593 (2)
P3—O1	1.476 (2)
P3—O2	1.479 (3)
P3—O9^ii^	1.594 (2)
P3—O8^iii^	1.597 (2)
Al—O3	1.852 (2)
Al—O2^iv^	1.873 (3)
Al—O1	1.877 (3)
Al—O4	1.887 (3)
Al—O5^v^	1.889 (3)
Al—O6^iii^	1.904 (2)

**Table:** 

P2^v^—O7—P1	139.91 (16)
P2—O8—P3^iii^	130.56 (16)
P1—O9—P3^vi^	129.42 (16)

Symmetry codes: (i) 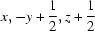; (ii) 
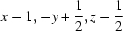
; (iii) 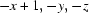; (iv) 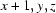; (v) 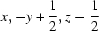; (vi) 
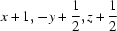
.

**Fig. 1.**
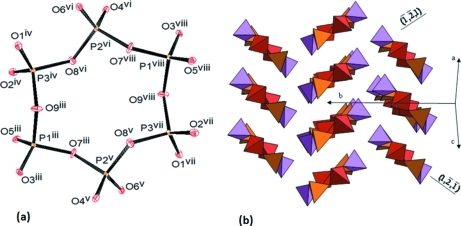
(a) ORTEP-3 view of the centrosymmetric (P6O18)6- ring anion. Displacement ellipsoids are drawn at the 50% probability level. Symmetry codes: (iii) 2 - x, 1/2 + y, 1/2 - z; (iv) 1 - x, 1 - y, -z; (v) 2 - x, 1 - y, 1 - z; (vi) x, 1 + y, z; (vii) 1 + x, 1 + y, 1 + z; (viii) x, 3/2 - y, 1/2 + z. (b) Partial projection along [101] showing the orientation of the mean planes of the (P6O18)6- ring anions.

**Fig. 2.**
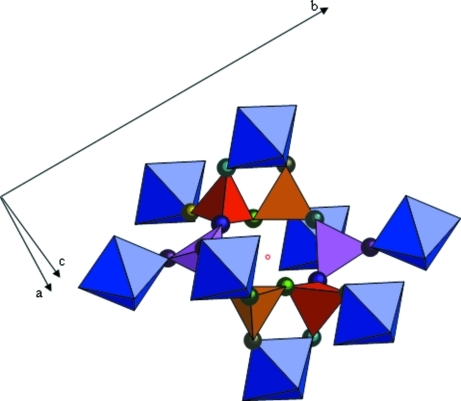
Partial projection showing the connection between the (P6O18)6- ring anion and the AlO6 octahedra.

## supplementary crystallographic information

### Comment

Although the Al_2_P_6_0_18_ cyclohexaphosphate is known since twenty five years (Kanene *et al.*, 1985), its crystal structure has never been refined from single crystal X-ray diffraction data. This paper deals with this purpose.

The crystal structure of Al_2_P_6_0_18_ is isotypic with Cr_2_P_6_O_18_ (Bagieu-Beucher & Guitel, 1977), Ga_2_P_6_O_18_ (Chudinova *et al.*, 1987) and Ru_2_P_6_O_18_ (Fukuoka *et al.*, 1995). It is built up of six-membered phosphate ring anions (P_6_O_18_)^6-^ isolated from each others and further linked by AlO_6_ octahedra by sharing corners. These centrosymmetric ring anions (P_6_O_18_)^6-^ are located around inversion centers at 0 0 1/2 and 0 1/2 0 (Fig. 1a). Their mean planes are parallel to either (121) or (121 ) planes (Fig. 1 b). Each AlO_6_ octahedron is linked to four P_6_O_18_ rings. More accurately, two rings are linked through bidentate diphosphate groups attached in *cis*-positions to the AlO_6_ octahedron. The two other rings are linked to the two remaining corners of the AlO_6_ octahedron (Fig. 2). Each (P_6_O_18_)^6-^ ring anion is connected to eight AlO_6_ octahedra by corner-sharing. Four diphosphate groups of the ring anion including P(3)O_4_ and either P(1)O_4_ or P(2)O_4_ tetrahedra are bidentate whereas the P(1)O_4_—P(2)O_4_ couple does not bind in a bidentate fashion. This may be correlated with the value of the P(1)—O—P(2) angle (139.91 (16)°) which is greater than P(2)—O—P(3) (130.56 (16)°) and P(1)—O—P(3) (129.42 (16) °) ones and also to the P(1)—P(2) distance (2.9741 (12) Å) slightly greater than P(2)—P(3) = 2.8970 (11) Å and P(1)—P(3) = 2.8826 (12) Å ones.

A survey of the internal symmetry of the (P_6_O_18_)^6-^ ring anions shows that most of them are centrosymmetric with P—P—P angles spreading from 87.8° to 142.8° (Averbuch-Pouchot & Durif, 1991*a*), i.e. with large deviations from the ideal value of 120°. When the (P_6_O_18_)^6-^ ring anion has internal 1 symmetry, it is built up of three independent P atoms and hence there are three characteristic α_i_ = P—P—P angles in the ring (α_1_ = P_1_—P_2_—P_3_, α_2_ = P_2_—P_3_—P_1_, α_3_ = P_3_—P_1_—P_2_). When taking the δ = Σ_i_|120-α_i_| parameter as a rough measure of the ring distorsion, Al_2_P_6_O_18_ exhibits the third lowest δ = 15.28° value after its homologous congeners Ru_2_P_6_O_18_ (δ = 13.78°) and Cr_2_P_6_O_18_ (δ = 14.39°). It should be noted that the characteristic P—P—P angles for both isotypic Ga_2_P_6_O_18_ and Fe_2_P_6_O_18_ structures have not been reported. The highest δ values calculated from data for other cyclohexaphosphates reported up to date are related to Cu_2_(NH_4_)_2_P_6_O_18_^.^8H_2_O (δ = 65.98°) (Averbuch-Pouchot & Durif, 1991*b*) and Ag_4_Li_2_P_6_O_18_.2H_2_O (δ = 67.29°) (Averbuch-Pouchot & Durif, 1991*c*).

For applications of aluminium phosphate, see: Vippola *et al.* (2000). For general reviews on the crystal chemistry of cyclohexaphosphates, see: Durif (1995) and Durif (2005).

### Experimental

Single crystals of the title compound have been obtained by reacting Al_2_O_3_ with (NH_4_)H_2_PO_4_ in an alumina boat. A mixture of these reagents in the molar ratio 1:6 was used for the synthesis. The mixture was first heated at 473 K for 24 h. Afterwards the temperature was successively raised to 573 K for 12 h, then to 673 K for 12 additional hours and finally to 923 K. After a heating period of 48 h at this temperature, the sample was cooled to room temperature by switching the furnace off. Translucent rhombs of Al_2_P_6_O_18_ were extracted from the batch.

### Refinement

The highest residual peak in the final difference Fourier map was located 0.47 Å from atom O7 and the deepest hole was located 1.12 Å from atom P3.

### Crystal data

**Table:**

Al_2_P_6_O_18_	*F*(000) = 520
*M**_r_* = 527.79	*D*_x_ = 2.412 Mg m^−^^3^
Monoclinic, *P*2_1_/*c*	Mo *K*α radiation, λ = 0.71073 Å
Hall symbol: -P 2ybc	Cell parameters from 2041 reflections
*a* = 6.0931 (2) Å	θ = 3.5–27.5°
*b* = 15.0676 (4) Å	µ = 0.96 mm^−^^1^
*c* = 8.2016 (3) Å	*T* = 296 K
β = 105.166 (1)°	Rhombic, colourless
*V* = 726.75 (4) Å^3^	0.16 × 0.07 × 0.06 mm
*Z* = 2	

### Data collection

**Table:**

Bruker APEXII CCD diffractometer	1674 independent reflections
Radiation source: fine-focus sealed tube	1398 reflections with *I* > 2σ(*I*)
graphite	*R*_int_ = 0.035
Detector resolution: 8.3333 pixels mm^-1^	θ_max_ = 27.5°, θ_min_ = 2.7°
φ and ω scans	*h* = −7→7
Absorption correction: multi-scan (*SADABS*; Bruker, 2008)	*k* = −11→19
*T*_min_ = 0.860, *T*_max_ = 0.945	*l* = −10→10
6548 measured reflections	

### Refinement

**Table:**

Refinement on *F*^2^	0 restraints
Least-squares matrix: full	Primary atom site location: structure-invariant direct methods
*R*[*F*^2^ > 2σ(*F*^2^)] = 0.032	Secondary atom site location: difference Fourier map
*wR*(*F*^2^) = 0.130	*w* = 1/[σ^2^(*F*_o_^2^) + (0.0787*P*)^2^ + 0.2264*P*] where *P* = (*F*_o_^2^ + 2*F*_c_^2^)/3
*S* = 1.16	(Δ/σ)_max_ < 0.001
1674 reflections	Δρ_max_ = 0.56 e Å^−^^3^
118 parameters	Δρ_min_ = −0.71 e Å^−^^3^

### Special details

**Table:**

Geometry. All esds (except the esd in the dihedral angle between two l.s. planes) are estimated using the full covariance matrix. The cell esds are taken into account individually in the estimation of esds in distances, angles and torsion angles; correlations between esds in cell parameters are only used when they are defined by crystal symmetry. An approximate (isotropic) treatment of cell esds is used for estimating esds involving l.s. planes.
Refinement. Refinement of *F*^2^ against ALL reflections. The weighted *R*-factor wR and goodness of fit *S* are based on *F*^2^, conventional *R*-factors *R* are based on *F*, with *F* set to zero for negative *F*^2^. The threshold expression of *F*^2^ > σ(*F*^2^) is used only for calculating *R*-factors(gt) etc. and is not relevant to the choice of reflections for refinement. *R*-factors based on *F*^2^ are statistically about twice as large as those based on *F*, and *R*- factors based on ALL data will be even larger.

### Fractional atomic coordinates and isotropic or equivalent isotropic displacement parameters (Å^2^)

**Table:**

	*x*	*y*	*z*	*U*_iso_*/*U*_eq_	
P1	0.70149 (15)	0.33738 (5)	0.01706 (11)	0.0049 (2)	
P2	0.64525 (14)	0.04431 (5)	0.21869 (11)	0.0050 (2)	
P3	0.08497 (14)	0.11301 (5)	−0.20937 (11)	0.0054 (2)	
Al	0.61549 (17)	0.13831 (6)	−0.12313 (13)	0.0046 (3)	
O1	0.3051 (4)	0.15855 (15)	−0.1454 (3)	0.0082 (5)	
O2	−0.0766 (4)	0.11484 (16)	−0.1026 (3)	0.0090 (5)	
O3	0.6755 (4)	0.25850 (15)	−0.0920 (3)	0.0078 (5)	
O4	0.6653 (4)	0.12162 (15)	0.1117 (3)	0.0076 (5)	
O5	0.5656 (4)	0.34555 (16)	0.1415 (3)	0.0084 (5)	
O6	0.4448 (4)	−0.01495 (15)	0.1608 (3)	0.0073 (5)	
O7	0.6509 (5)	0.42395 (15)	−0.0967 (3)	0.0109 (5)	
O8	0.8737 (4)	−0.01227 (15)	0.2539 (3)	0.0110 (5)	
O9	0.9653 (4)	0.34779 (16)	0.1085 (3)	0.0107 (5)	

### Atomic displacement parameters (Å^2^)

**Table:**

	*U*^11^	*U*^22^	*U*^33^	*U*^12^	*U*^13^	*U*^23^
P1	0.0052 (5)	0.0050 (4)	0.0042 (5)	−0.0007 (3)	0.0008 (3)	0.0001 (3)
P2	0.0060 (5)	0.0041 (4)	0.0050 (5)	−0.0003 (3)	0.0014 (3)	−0.0009 (3)
P3	0.0036 (5)	0.0058 (4)	0.0069 (5)	0.0007 (3)	0.0015 (3)	0.0002 (3)
Al	0.0034 (5)	0.0055 (5)	0.0052 (5)	−0.0006 (4)	0.0015 (4)	−0.0003 (3)
O1	0.0045 (12)	0.0065 (12)	0.0136 (13)	−0.0012 (9)	0.0025 (10)	−0.0021 (9)
O2	0.0050 (12)	0.0127 (12)	0.0094 (13)	−0.0002 (9)	0.0024 (10)	0.0014 (10)
O3	0.0110 (13)	0.0045 (11)	0.0077 (12)	−0.0028 (9)	0.0020 (10)	−0.0015 (9)
O4	0.0119 (13)	0.0059 (11)	0.0053 (12)	−0.0022 (9)	0.0025 (10)	−0.0007 (9)
O5	0.0066 (13)	0.0132 (12)	0.0057 (12)	0.0000 (9)	0.0020 (10)	0.0003 (9)
O6	0.0060 (12)	0.0063 (12)	0.0098 (12)	0.0002 (9)	0.0025 (10)	−0.0012 (9)
O7	0.0221 (15)	0.0059 (12)	0.0053 (12)	0.0033 (9)	0.0045 (11)	0.0022 (9)
O8	0.0061 (12)	0.0073 (12)	0.0175 (14)	−0.0004 (9)	−0.0007 (10)	−0.0038 (9)
O9	0.0052 (13)	0.0186 (13)	0.0084 (13)	−0.0021 (10)	0.0019 (10)	−0.0062 (10)

### Geometric parameters (Å, °)

**Table:**

P1—O3	1.471 (2)	Al—O3	1.852 (2)
P1—O5	1.478 (2)	Al—O2^iv^	1.873 (3)
P1—O7	1.587 (2)	Al—O1	1.877 (3)
P1—O9	1.594 (3)	Al—O4	1.887 (3)
P2—O4	1.482 (2)	Al—O5^v^	1.889 (3)
P2—O6	1.487 (2)	Al—O6^iii^	1.904 (2)
P2—O7^i^	1.579 (2)	O2—Al^vi^	1.873 (3)
P2—O8	1.593 (2)	O5—Al^i^	1.889 (3)
P3—O1	1.476 (2)	O6—Al^iii^	1.904 (2)
P3—O2	1.479 (3)	O7—P2^v^	1.579 (2)
P3—O9^ii^	1.594 (2)	O8—P3^iii^	1.597 (2)
P3—O8^iii^	1.597 (2)	O9—P3^vii^	1.594 (3)
			
O3—P1—O5	119.77 (14)	O3—Al—O4	90.93 (11)
O3—P1—O7	109.47 (14)	O2^iv^—Al—O4	89.61 (11)
O5—P1—O7	106.27 (14)	O1—Al—O4	90.56 (12)
O3—P1—O9	107.50 (14)	O3—Al—O5^v^	89.33 (11)
O5—P1—O9	110.17 (14)	O2^iv^—Al—O5^v^	90.32 (11)
O7—P1—O9	102.27 (14)	O1—Al—O5^v^	89.50 (11)
O4—P2—O6	118.09 (14)	O4—Al—O5^v^	179.74 (12)
O4—P2—O7^i^	110.17 (13)	O3—Al—O6^iii^	178.54 (12)
O6—P2—O7^i^	107.36 (14)	O2^iv^—Al—O6^iii^	88.66 (11)
O4—P2—O8	108.93 (14)	O1—Al—O6^iii^	89.77 (11)
O6—P2—O8	110.01 (13)	O4—Al—O6^iii^	90.46 (11)
O7^i^—P2—O8	100.91 (14)	O5^v^—Al—O6^iii^	89.29 (11)
O1—P3—O2	117.68 (15)	P3—O1—Al	139.30 (16)
O1—P3—O9^ii^	108.14 (14)	P3—O2—Al^vi^	139.15 (17)
O2—P3—O9^ii^	109.62 (14)	P1—O3—Al	149.33 (16)
O1—P3—O8^iii^	109.89 (14)	P2—O4—Al	133.84 (15)
O2—P3—O8^iii^	108.82 (14)	P1—O5—Al^i^	138.32 (16)
O9^ii^—P3—O8^iii^	101.47 (14)	P2—O6—Al^iii^	138.20 (15)
O3—Al—O2^iv^	90.88 (11)	P2^v^—O7—P1	139.91 (16)
O3—Al—O1	90.69 (11)	P2—O8—P3^iii^	130.56 (16)
O2^iv^—Al—O1	178.43 (11)	P1—O9—P3^vii^	129.42 (16)

Symmetry codes: (i) *x*, −*y*+1/2, *z*+1/2; (ii) *x*−1, −*y*+1/2, *z*−1/2; (iii) −*x*+1, −*y*, −*z*; (iv) *x*+1, *y*, *z*; (v) *x*, −*y*+1/2, *z*−1/2; (vi) *x*−1, *y*, *z*; (vii) *x*+1, −*y*+1/2, *z*+1/2.
